# Explanatory factors for first and second-generation non-western women’s inadequate prenatal care utilisation: a prospective cohort study

**DOI:** 10.1186/s12884-015-0528-x

**Published:** 2015-04-21

**Authors:** Agatha W Boerleider, Judith Manniën, Cherelle MV van Stenus, Therese A Wiegers, Esther I Feijen-de Jong, Evelien R Spelten, Walter LJM Devillé

**Affiliations:** Netherlands Institute for Health Services Research (NIVEL), PO Box 1568, 3500 BN Utrecht, the Netherlands; Department of Midwifery Science, AVAG and the EMGO Institute for Health and Care Research, VU University Medical Center, Amsterdam, The Netherlands; Athena Institute for Research on Innovation and Communication in Health and Life Sciences, VU University, Amsterdam, the Netherlands; Faculty of Medicine, Nursing and Health Sciences, Monash University, Melbourne, Australia; Faculty of Social and Behavioural Sciences, University of Amsterdam, Amsterdam, the Netherlands; National Knowledge and Advisory Centre on Migrants, Refugees and Health (Pharos), Utrecht, the Netherlands

**Keywords:** Prenatal care, Care utilisation, Non-western, Immigrants, Generation

## Abstract

**Background:**

Little research into non-western women’s prenatal care utilisation in industrialised western countries has taken generational differences into account. In this study we examined non-western women’s prenatal care utilisation and its explanatory factors according to generational status.

**Methods:**

Data from 3300 women participating in a prospective cohort of primary midwifery care clients (i.e. women with no complications or no increased risk for complications during pregnancy, childbirth and the puerperium who receive maternity care by autonomous midwives) in the Netherlands (the DELIVER study) was used. Gestational age at entry and the total number of prenatal visits were aggregated into an index. The extent to which potential factors explained non-western women’s prenatal care utilisation was assessed by means of blockwise logistic regression analyses and percentage changes in odds ratios.

**Results:**

The unadjusted odds of first and second-generation non-western women making inadequate use of prenatal care were 3.26 and 1.96 times greater than for native Dutch women. For the first generation, sociocultural factors explained 43% of inadequate prenatal care utilisation, socioeconomic factors explained 33% and demographic and pregnancy factors explained 29%. For the second generation, sociocultural factors explained 66% of inadequate prenatal care utilisation.

**Conclusion:**

Irrespective of generation, strategies to improve utilisation should focus on those with the following sociocultural characteristics (not speaking Dutch at home, no partner or a first-generation non-Dutch partner). For the first generation, strategies should also focus on those with the following demographic, pregnancy and socioeconomic characteristics (aged ≤19 or ≥36, unplanned pregnancies, poor obstetric histories (extra-uterine pregnancy, molar pregnancy or abortion), a low educational level, below average net household income and no supplementary insurance.

**Electronic supplementary material:**

The online version of this article (doi:10.1186/s12884-015-0528-x) contains supplementary material, which is available to authorized users.

## Background

Prenatal care provides an opportunity to address pregnancy complications, promote a healthy lifestyle and prepare women and their families for the birth and parenting [1]. The benefits of early prenatal care entry are well recognised and supported by studies which have demonstrated an association between late prenatal care entry and adverse pregnancy outcomes [2,3]. A Cochrane review, which was updated in 2010, showed that women in prenatal care programmes with reduced numbers of visits had a borderline significant increase in perinatal mortality compared to women receiving standard prenatal care (RR 1.14; 95% CI 1.00 - 1.31) [4]. However, the ideal number of prenatal visits has been much debated [5,6].

In most industrialised western countries, prenatal care is universally accessible. Nevertheless, previous research suggests that certain groups of women (including non-western women (i.e. women originating from Asia, Africa, Latin America and Turkey whose socioeconomic position and sociocultural values differ from that of the majority populations in industrialised western countries)) are more likely to make inadequate use of prenatal care, i.e. late entry and /or an insufficient number of visits. In the United Kingdom, Asian and black women were found to be more likely to enter prenatal care late than white women [7-9]. Pakistani and Indian women were found to attend significantly fewer prenatal care visits than native British women [10]. In Australia, migrants from developing countries were more likely to enter prenatal care late than non-Aboriginal Australian-born women (OR 2.18; 95% CI 2.1 - 2.26) [11]. However, non-western ethnicity does not of itself explain these women’s inadequate utilisation of prenatal care; instead, via a number of underlying factors, it influences the need, propensity and ability to make use of care [12,13].

Quantitative research specifically aimed at exploring the factors behind non-western women’s prenatal care utilisation in industrialised western countries with universally accessible healthcare is limited. Three studies coincidentally all conducted in Dutch urban regions, have reported some explanatory factors for non-western women’s late entry and/or insufficient number of visits [14-16]. This raises the question whether these findings are limited to urban regions and to which extent the reported factors explain non-western women’s inadequate prenatal care utilisation.

Recently, another regional Dutch study has yielded new insights [17]. First-generation non-western women are more likely to enter prenatal care late than those of the second generation. This delay is explained primarily by a less active attitude towards healthy behaviour. This new insight raises the question of whether generational differences also exist in overall prenatal care utilisation. In this national study we therefore wanted to explore first and second-generation non-western women’s prenatal care utilisation, taking not only the gestational age at entry but also the numbers of visits into account. By comparing their prenatal care utilisation to that of native Dutch women, we want to allow for comparison of our results with those of previous conducted studies. Based on these aims the following research question was formulated: How do first and second-generation non-western women utilise prenatal care compared to native Dutch women and – if there is a difference – what factors can explain this?

## Methods

### Data collection

Data from the national DELIVER study, a multi-centre prospective dynamic cohort study that aimed to evaluate the quality, organisation and accessibility of primary midwifery care in the Netherlands, was used for this study [18]. Primary midwifery care is maternity care provided by autonomous midwives to women with no complications or no increased risk for complications during pregnancy, childbirth and the puerperium. Of the 2852 practicing midwives in the Netherlands on the first of January 2013, 1621 - one per 10.000 women within the age range 15 to 39 - worked in primary care [19]. Between 1999 and 2008, 83.1% of the pregnant women in the Netherlands had their first prenatal appointment at a primary care midwife. Eventually, 35.8% gave birth under supervision of a primary care midwife [20].

The cohort consisted of primary midwifery care clients who had completed up to three questionnaires between their first prenatal appointment and six weeks postpartum. Clients could enrol at any moment in their pregnancy and were invited to respond to the appropriate questionnaire. The first or early prenatal questionnaire was completed before 35 weeks of gestation, the second or late prenatal questionnaire between 35 weeks of gestation and birth, and the third or postpartum questionnaire around 6 weeks postpartum. By completing a questionnaire, clients implicitly gave consent to participate in the study. Ethical approval was obtained from the medical ethics committee of the VU University Medical Center in the Netherlands (WC 008–100).

The women in this cohort were recruited between September 2009 and February 2011 by the midwives from 20 midwifery practices spread all over the country. These midwifery practices were selected according to three stratification criteria: region, level of urbanisation and practice type. A total of 14148 clients were invited to participate. Of these, 12398 were eligible. The response rate for at least one questionnaire was 62% [18]. For each participating client, questionnaire data was linked to data from the national Netherlands Perinatal Registry and the electronic client record in the midwifery practices by means of unique anonymous identifiers for the client and midwifery practice.

### Study population

This study included native Dutch and non-western women (see the definitions in the description of the main independent variable) who had completed the early prenatal questionnaire of the DELIVER study. Because of our specific interest in non-western women’s prenatal care utilisation compared to that of native Dutch women, migrant women of western origin were excluded. From the literature it was obvious that non-western women were at higher risk for inadequate use. Furthermore, women lacking additionally linked data and women who did not start prenatal care with a primary care midwife were excluded.

**Figure 1:**
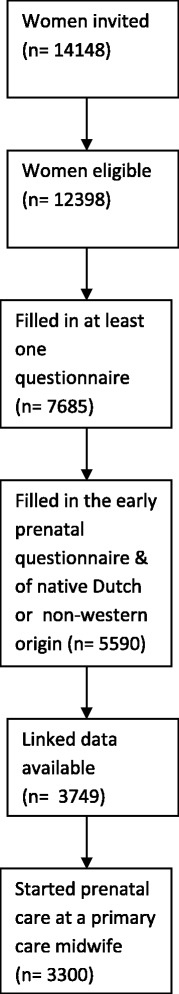
Schematic draft of the selection process of the study population.

Figure 1 shows a schematic draft of the selection process of the study population. Of the 5590 native Dutch and non-western women who had completed the early prenatal questionnaire of the DELIVER study, 3749 (67.1%) had additionally linked data on prenatal care utilisation. Of these, 3300 women had started prenatal care with a primary care midwife. Table 1 shows the characteristics of these 3300 women according to ethnic origin.

**Table 1:** **Characteristics of the study population (N = 3300)**

	**Native Dutch**	**First-generation non-western**	**Second-generation non-western**
	**(N = 2998)**	**(N = 203)**	**(N = 99)**
	**N (%)**	**N (%)**	**N (%)**
Prenatal care utilisation			
Adequate	2764 (92.2%)	158 (77.8%)	83 (83.8%)
Inadequate	234 (7.8%)	45 (22.2%)	16 (16.2%)
- entered on time, but insufficient number of visits	38 (1.3%)	3(1.5%)	2 (2.0%)
- entered late	196 (6.5%)	42 (20.7%)	14 (14.1%)
Age			
<= 19	25 (0.8%)	2 (1.0%)	2 (2.0%)
20-35	2545 (85.0%)	174 (86.1%)	93 (93.9%)
> = 36	425 (14.2%)	26 (12.9%)	4 (4.0%)
Parity			
Nulliparous	1262 (42.1%)	65 (32.3%)	45 (45.5%)
Primi-/multiparous	1735 (57.9%)	136 (67.7%)	54 (54.5%)
Educational level			
High	1461 (48.8%)	63 (31.2%)	41 (41.8%)
Medium	1104 (36.9%)	59 (29.2%)	37 (37.8%)
Low	428 (14.3%)	80 (39.6%)	20 (20.4%)
Net household income			
Average	629 (21.1%)	32 (16.1%)	20 (20.6%)
Below average	381 (12.8%)	101 (50.8%)	26 (26.8%)
Above average	1452 (48.6%)	34 (17.1%)	37 (38.1%)
Did not want to say	524 (17.5%)	32 (16.1%)	14 (14.4%)

Of the 2998 native Dutch women, 234 (7.8%) made inadequate use of prenatal care – 6.5% entering late and 1.3% with an insufficient number of visits. Of the 203 first-generation non-western women, 45 (22.2%) made inadequate use of prenatal care – 20.7% entering late and 1.5% with an insufficient number of visits. Of the 99 second-generation non-western women, 16 (16.2%) made inadequate use of prenatal care – 14.1% entering late and 2.0% with an insufficient number of visits.

Table 2 shows the ethnic origin of the 302 non-western women. The majority (64.9%) belonged to one of the four major non-western groups in the Netherlands: Turkish, Moroccan, Surinamese, Dutch Antillean/Aruban. Turkey and Morocco supplied the Netherlands with large numbers of guest workers during the economic growth in the 60’s and 70’s. Suriname and the Dutch Antilles/Aruba are former Dutch colonies.

**Table 2:** **Ethnic origin of the non-western population (N = 302)**

	**N (%)**
Turkish	67 (22.2%)
Moroccan	80 (26.5%)
Surinamese	32 (10.6%)
Dutch Antillean/Aruban	17 (5.6%)
Other	106 (35.1%)

### Dependent variable

An index was compiled to assess women’s prenatal care utilisation as comprehensive as possible by taking both prenatal care entry and the number of prenatal visits into account (see Additional file 1). This index was derived from the Kotelchuck index [21], and adapted to the Dutch primary midwifery care context.

Prenatal care entry was determined based on the gestational age at the first prenatal visit (derived from the ultrasound scan or the first day of the last menstrual period) and classified into ‘on time’ (gestational age at onset < 12 weeks) and ‘late’ (gestational age at onset ≥ 12 weeks).

The number of prenatal visits was derived from the electronic client record, and compared to the expected number of visits derived from the guidelines of the Royal Dutch Organisation of Midwives (KNOV) [22]. The expected number of prenatal visits was based on the gestational age at which women gave birth. For women who were referred to secondary care, the expected number of prenatal visits was based on the gestational age at referral.

For this study, the modified index (see Additional file 1) was dichotomised into:

1. Inadequate utilisation: onset at ≥ 12 weeks *and/or* received < 50% of expected visits
2. Adequate utilisation: onset < 12 weeks *and* received ≥ 50% of expected visits. (This category also includes women who made more than adequate use of prenatal care (i.e. received ≥ 110% of expected visits)

### Main independent variable

The main independent variable was women’s ethnicity, which was categorised into native Dutch, first-generation non-western and second-generation non-western. Ethnicity was established by asking women to fill in their country of birth and their parents’ in the questionnaire. The classification into native Dutch and non-western was made according to the definition used by Statistics Netherlands [23]. Women are considered native Dutch when both of their parents were born in the Netherlands, and non-western when at least one of their parents was born in Asia (excluding Indonesia and Japan), Africa, Latin America or Turkey. Non-western women were subdivided into first generation (born outside the Netherlands) and second generation (born in the Netherlands).

### (Potential) Explanatory independent variables

Based on Andersen’s healthcare utilisation model [24] and the conceptual framework of Foets *et al.* [12], several factors were considered as potential explanatory variables for the association between ethnicity and prenatal care utilisation. These variables were derived from the early prenatal questionnaire of the DELIVER study and assigned to the following blocks of conceptually linked variables:

- *Demographic and pregnancy factors*: maternal age (≤19, 20–35, ≥ 36 years); parity (nulliparous, primi-/multiparous); pregnancy intention (planned and wanted, unplanned but wanted, unplanned and unwanted); an ectopic pregnancy, molar pregnancy or abortion in the obstetric history (no, yes).
- *Socioeconomic factors*: level of maternal education (high, medium, low); net household income (average, below average, above average, won’t say); supplementary insurance (no, yes).
- *Sociocultural factors:* partner’s ethnicity (native Dutch, first-generation non-Dutch, second- generation non-Dutch, no partner); language spoken at home (Dutch or other).
- *Psychological factors:* perceived locus of control concerning their own health (a lot, no or hardly); perceived health status (good, poor); fear of giving birth (not so anxious, anxious,); fear of bearing a handicapped child (not so anxious, anxious); pregnancy-related concerns about their appearance (not so anxious, anxious,).
- *Health behaviour:* currently smoking (no, yes); alcohol use since discovering pregnancy (no, yes); folic acid use (yes, no); Body Mass Index (BMI )(not overweight (<25), overweight (25–30), obese (≥30)).
- *Accessibility of the midwifery practice:* calling the practice (no problem or never tried, problem); visiting the practice (no problem, problem); booking appointments with the practice (no problem, problem).

### Data analysis

Data analysis consisted of several stages. Firstly, descriptive analyses were carried out on the independent and dependent variables. Secondly, univariable logistic regression analyses were carried out to determine the association between the potential explanatory independent variables and the dependent variable. Only potential explanatory independent variables associated with the dependent variable (p < 0.25) were retained in the corresponding category for further analyses. Thirdly, blockwise multivariable logistic regression analyses were conducted, because of the dichotomous outcome measure and the focus on groups of explanatory factors [25]. The first block consisted only of the main independent variable, namely ethnicity. After this, six separate logistic regression analyses were conducted, adjusting each time for one block of explanatory variables. In addition to these separate logistic regression analyses, a blockwise structured model was constructed by adding the blocks of explanatory independent variables one by one. The blocks of explanatory variables most proximate to the individual were added first and those most distant last, until a final model consisting of the main independent variable and all six blocks was constructed. Besides these ordinary univariable and blockwise mutltivariable logistic regression analyses we also conducted univariable and blockwise multivariable multilevel logistic regression analyses. Latter were conducted to take the hierarchical nature of the data, i.e. clients (level 1) nested within midwifery practices (level 2) into account. If the multilevel model resulted in a significantly better fit than the ordinary univariable or blockwise multivariable logistic regression model, the former was preferred and presented. Lastly, the percentage change in odds ratio (OR) was calculated for each model, using the following formula: (((OR _(model 2–13)_ - OR _(model 1)_)/(OR _(model 1)_ - 1)) ×100) [26].

Using the percentage changes in OR, the overall and direct contributions of each block of explanatory variables to inadequate prenatal care utilisation were calculated with the following formulas:

1. Overall effect (x) = percentage change in OR from the separate block wise analysis (x)
2. Direct effect (x) = [percentage change in OR from the blockwise structured model (x)] - [percentage change in OR from the blockwise structured model (x-1)];

All analyses were conducted in IBM SPSS Statistics (version 20.0), except for the multilevel analyses which were conducted in Stata (version 12).

## Results

### Explanatory independent variables

Table 3 shows the results of the univariable analysis. Fifteen of the 21 potential explanatory variables were significantly associated with prenatal care utilisation and retained in the corresponding category for use in the blockwise multivariable logistic regression analyses. The potential explanatory variables excluded from further analysis were alcohol use, fear of bearing a handicapped child, pregnancy-related concerns about appearance, perceived health status and accessibility of the midwifery practice (calling the practice and visiting the practice).

**Table 3:** **Association between the potential explanatory independent variables and inadequate prenatal care utilisation (assessed by multilevel univariable logistic regression analyses) (N = 3300)**

	**Odds ratio**	**95% CI**	**p Value**
**Demographic and pregnancy factors**			
**Age**			
<= 19	5.30	2.15 to 13.05	0.00
20-35*	1		
> = 36	1.57	1.12 to 2.18	0.01
**Parity**			
Nulliparous*	1		
Primi-/multiparous	0.843	0.65 to 1.09	0.19
**Pregnancy intention**			
Planned and wanted*	1		
Unplanned but wanted	1.91	1.41 to 2.59	0.00
Unplanned and unwanted	44.47	7.92 to 249.75	0.00
**Obstetric history**			
(extra-uterine pregnancy, molar pregnancy or abortion)			
No*	1		
Yes	1.53	1.04 to 2.24	0.03
**Socioeconomic factors**			
**Educational level**			
High*	1		
Medium	1.07	0.80 to 1.45	0.64
Low	1.92	1.36 to 2.70	0.00
**Net household income**			
Average*	1		
Below average	1.64	1.11 to 2.44	0.01
Above average	0.73	0.51 to 1.04	0.08
Did not want to say	1.20	0.80 to 1.80	0.37
**Supplementary insurance**			
Yes*	1		
No	1.51	1.07 to 2.14	0.02
**Sociocultural factors**			
**Partner**			
Native Dutch*	1		
First-generation non-Dutch	3.27	2.23 to 4.80	0.00
Second-generation non-Dutch	1.23	0.7 to 2.07	0.43
No partner	4.87	2.51 to 9.48	0.00
**Language spoken at home**			
Dutch*	1		
Other	3.73	2.48 to 5.61	0.00
**Psychological factors**			
**Perceived locus of control concerning their own health**			
A lot*	1		
No or hardly	1.60	1.16 to 2.20	0.00
Perceived health status			
Good*	1		
Poor	1.10	0.83 to 1.47	0.50
**Fear of giving birth**			
Less anxious*	1		
Anxious	1.28	0.98 to 1.66	0.07
Fear of bearing a handicapped child			
Less anxious*	1		
Anxious	0.86	0.66 to 1.13	0.29
Pregnancy-related concerns about their appearance			
Less anxious*	1		
Anxious	0.99	0.75 to 1.30	0.94
**Health behaviour factors**			
**Smoking**			
No*	1		
Yes	1.36	0.90 to 2.04	0.14
Alcohol			
No*	1		
Yes	0.99	0.65 to 1.49	0.96
**Folic acid**			
Yes*	1		
No	3.99	2.85 to 5.57	0.00
**Body mass index**			
Not overweight*	1		
Overweight	1.18	0.85 to 1.64	0.32
Obese	1.63	1.06 to 2.49	0.02
**Accessibility of the midwifery practice**			
Calling the midwifery practice			
No problem or never tried*	1		
Problem	1.00	0.71 to 1.40	0.98
Visiting the midwifery practice			
No problem*	1		
Problem	1.38	0.75 to 2.54	0.30
**Booking appointments with the midwifery practice**			
No problem*	1		
Problem	2.03	1.24 to 3.33	0.01

* = Reference category.

Significance level: p <0.25.

Variables in bold were significantly associated with prenatal care utilisation and retained in the corresponding category for the multilevel block wise logistic regression analysis.

### First and second-generation non-western women’s prenatal care utilisation compared to native Dutch women

Table 4 shows the results of the multivariable logistic regression analyses. The unadjusted odds of first and second-generation non-western women making inadequate use of prenatal care are 3.26 (95% CI 2.13 - 5.00) and 1.96 (95% CI 1.08 - 3.57) times greater than for native Dutch women.

**Table 4:** **Unadjusted and adjusted odds ratios, 95% confidence intervals (95% C.I.) and percentage change in odds ratios for inadequate use of prenatal care according to generation (assessed by multilevel blockwise logistic regression analyses; reference category: native Dutch women)**

	**First-generation non-western**			**Second-generation non-western**		
	**OR**	**95% CI**	**% change**	**OR**	**95% CI**	**% change**
1. Unadjusted	3.26	2.13 to 5.00		1.96	1.08 to 3.57	
Separate blockwise analyses:						
2. Adjusted for demographic and pregnancy factors	2.61	1.65 to 4.13	−29%	1.94	1.04 to 3.59	−2%
3. Adjusted for socioeconomic factors	2.13	1.32 to 3.43	−50%	1.78	0.97 to 3.27	−19%
4. Adjusted for sociocultural factors	1.14	0.59 to 2.18	−94%	1.10	0.55 to 2.22	−90%
5. Adjusted for psychological factors	2.94	1.87 to 4.62	−14%	1.74	0.93 to 3.23	−23%
6. Adjusted for health behavioural factors	1.80	1.08 to 3.02	−65%	1.79	0.94 to 3.42	−18%
7. Adjusted for accessibility factors	3.31	2.16 to 5.08	2%	1.99	1.09 to 3.63	3%
Blockwise structured model:						
8. Adjusted for demographic and pregnancy factors	2.61	1.65 to 4.13	−29%	1.94	1.04 to 3.59	−2%
9. Model 8 & socioeconomic factors	1.87	1.12 to 3.10	−62%	1.86	1.00 to 3.47	−10%
10. Model 9 & sociocultural factors	0.89	0.44 to 1.83	−105%	1.23	0.61 to 2.50	−76%
11. Model 10 & psychological factors	0.93	0.45 to 1.91	−103%	1.21	0.58 to 2.50	−78%
12. Model 11 & health behavioural factors	0.62	0.27 to 1.40	−117%	1.11	0.51 to 2.44	−89%
13. Model 12 & accessibility factors	0.64	0.28 to 1.45	−116%	1.12	0.51 to 2.47	−88%

Read % changes higher than 100% as 100%.

In the *separate blockwise analyses*, the percentage changes in odds ratios demonstrate that first and second-generation non-western women’s inadequate prenatal care utilisation could largely be explained by the overall effects of sociocultural factors (at 94% and 90% respectively) and to a lesser extent by health behaviour factors (65% and 18% respectively) and socioeconomic factors (50% and 19% respectively). In addition to these corresponding explanatory factors, the overall effects of demographic and pregnancy factors explained 29% of first-generation women’s inadequate prenatal care utilisation, and the overall effects of psychological factors 23% of that of second-generation women.

In the *blockwise structured model*, adjusting first for demographic and pregnancy factors, then for socioeconomic factors and then for sociocultural factors resulted in a continued reduction of both first and second-generation non-western women’s higher odds of inadequate prenatal care utilisation to that of native Dutch women. After adjusting for these three blocks of factors, 105% and 76% of first and second-generation non-western women’s inadequate prenatal care utilisation respectively could be explained. The percentage changes in odds ratios demonstrate that first and second-generation non-western women’s inadequate prenatal care utilisation could largely be explained by the direct effects of sociocultural factors, at 43% (105%-62%) and 66% (76%-10%) respectively. For first-generation women, the direct effects of two other blocks of factors also explained a large part of their inadequate prenatal care utilisation: socioeconomic factors, at 33% (62%-29%), and demographic and pregnancy factors, at 29%.

## Discussion

Up to now, very few studies have examined non-western women’s prenatal care utilisation according to generational status. In our study, both first and second-generation women were more likely to make inadequate use of prenatal care compared to native Dutch women, mainly as a result of late entry. After adjusting concurrently for all 6 blocks of explanatory variables, the difference in prenatal care utilisation for first and second-generation non-western women compared to native Dutch women, could be explained fully and nearly fully (88%) respectively. This lower percentage for the second generation indicates that other factors besides those included in our study may play a minor role in the explanation of their prenatal care utilisation.

There were not only similarities but also differences in the explanatory factors for first and second-generation non-western women. Sociocultural factors contributed substantially, irrespective of generation. Socioeconomic, demographic and pregnancy factors also contributed substantially, but only for first-generation women. Explanatory factor for this difference between the first and second generations may include the second generation’s higher educational level and better financial situation [27].

The major contribution from sociocultural factors sheds new light on non-western women’s prenatal care utilisation. In a qualitative study conducted in Australia, Thai women whose husband spoke the official language reported that their husbands arranged prenatal care [28]. Poor language proficiency has previously been reported to be an explanatory factor for non-western women’s prenatal care entry [14]. In this study we were able to quantitatively explore this further by including the ethnic origin of the partner and the language spoken at home as sociocultural factors. Our study revealed that these factors provide the bulk of the explanation of non-western women’s inadequate prenatal care utilisation. However, it should not be overlooked that the language spoken at home was used as a surrogate of women’s Dutch language proficiency. It might be possible that some women with good Dutch language proficiency still speak another language at home.

The substantial contribution of socioeconomic factors to the explanation of first-generation non-western women’s inadequate prenatal care utilisation corresponds to findings of previously conducted studies, which reported low maternal education [14,15] and not having a paid job [15] as factors explaining at least part of non-western women’s late prenatal care entry. The universal accessibility of prenatal care in the Netherlands makes this substantial contribution an interesting finding. This finding may perhaps reflect first-generation women’s limited knowledge about the organisation of the Dutch prenatal care system (e.g. at what gestational age to enter prenatal care) or their daily occupations and concerns.

Demographic and pregnancy factors also contributed substantially to the explanation of first-generation non-western women’s inadequate prenatal care utilisation. This corresponds to the results of previous studies, which have reported multiparity [14]; having an unplanned and unwanted pregnancy [14]; and young age [14] as factors explaining non-western women’s late prenatal care entry. To gain a better understanding, the mechanisms by which some of these separate factors may affect prenatal care utilisation need to be explored further, for instance, the possible reasons behind multiparous women’s inadequate prenatal care utilisation, such as less appreciation of prenatal care or the lack of childcare for the other children [13].

Our findings also show similarities to those of a systematic review on determinants of inadequate prenatal care utilisation in industrialised western countries [29]. This review, which did not specifically focus on non-western women, also reported socioeconomic factors (e.g. low educational level, living in neighbourhoods with higher rates of unemployment) and demographic and pregnancy factors (e.g. high parity) as determinants. This demonstrates that these groups of factors are not unique to non-western women. However, our sociocultural variables (i.e. language spoken at home, origin of partner) were not found in this review, and therefore do seem to relate more specifically to non-western women in industrialised western countries.

Contrary to previous research findings that pointed to folic acid use as an explanatory factor [17], the block of health behaviour factors was not found to contribute substantially as an explanatory factor in our study. The large overall effect of health behaviour factors in the separate blockwise model, but small direct effect in the blockwise structured model indicates that they mainly exert their effect on prenatal care utilisation indirectly through other factors.

The blocks with psychological and accessibility factors contributed the least, even though many new potential explanatory variables were included. It should be noted that in the blockwise structured model, these blocks of variables were added to the model last. This may have led to an underestimation of their direct effect. However, their overall effect in the separate blockwise model was also minimal.

The index used to measure prenatal care utilisation was adjusted to the guidelines of the Royal Dutch Organisation of Midwives (KNOV), and is therefore only applicable in the Dutch primary midwifery care context. Furthermore, this index only measured prenatal care utilisation (the gestational age at entry and the number of prenatal visits), and did not take the content of prenatal care into account. However, adequate utilisation of prenatal care does not necessarily mean that women have received prenatal care of adequate content. The quality of prenatal care received, and compliance to recommended prenatal care standards by their prenatal care provider may be below standard. Therefore, future studies combining the adequacy of the content of prenatal care and the adequacy of prenatal care utilisation are recommended. A tool assessing these two prenatal care aspects has already been developed and applied in scientific research [30,31].

In a previous conducted study by Choté *et al*., non-western women’s inadequate prenatal care utilisation was mainly characterised by late entry and much less by an insufficient number of visits [16]. Our study adds to this existing knowledge by revealing a predominant role of late entry among first and second-generation non-western women making inadequate use of prenatal care. The difference in explanatory factors for first and second-generation non-western women also adds to the knowledge of previous quantitative studies which have predominantly reported factors for specific non-western groups. Together these findings demonstrate hat prenatal care utilisation differs between generations and ethnic subgroups within the non-western population. Furthermore, this study confirms the findings of previously conducted qualitative studies which have reported lack of support from family [32], language proficiency [33-36], financial problems [36] and social inequalities [32] as factors impeding non-western women’s prenatal care utilisation.

### Strength and limitations

A major strength of this study is the national dataset containing a large number of variables. Another strength is the adjustment of the Kotelchuck index in line with the guidelines of the Royal Dutch Organisation of Midwives (KNOV). A Kotelchuck index adjusted to the guidelines of the Dutch Society of Obstetrics and Gynaecology (NVOG) already existed [12], but this seemed less appropriate as we were focusing on primary midwifery care. The selection and classification of the explanatory variables based on theoretical frameworks may also be considered as another strength. Despite of the strengths, some limitations need to be taken into account. Firstly, we were not able to distinguish different ethnic groups within the groups of first and second-generation non-western women because this would have resulted in small subgroups. Secondly, we were not able to compare first and second-generation non-western women’s prenatal care utilisation against each other, because the numbers were insufficient. Thirdly, the adjusted response rate of 62% with highly educated women being overrepresented in this sample (47.5%), may have led to an underestimation of the number of inadequate users and the contribution of socioeconomic factors.

## Conclusions

There are not only similarities but also differences in the explanatory factors for first and second-generation non-western women’s prenatal care utilisation. Irrespective of generation, strategies to improve utilisation should focus on women with the following sociocultural characteristics: not speaking Dutch at home, and having no partner or a first-generation non-Dutch partner. For the first generation, strategies should also focus on women with the following demographic, pregnancy and socioeconomic characteristics: age ≤ 19 or ≥36 years, unplanned pregnancy, poor obstetric history (extra-uterine pregnancy, molar pregnancy or abortion), low educational level, below average net household income and no supplementary insurance. These strategies include propagating the benefits of adequate prenatal care utilisation through community/cultural organisations and websites/leaflets of midwifery and general practices. Furthermore, midwives should keep a close eye on the prenatal care utilisation of women with these characteristics to enable timely intervention in case of inadequate utilisation. These findings underline the importance of taking generational differences into account when developing strategies to improve non-western women’s prenatal care utilisation. They are therefore also relevant to other western countries with substantial non-western populations, and need to be supported by more and larger studies exploring explanatory factors within subgroups of first and second-generation non-western women (e.g. first and second generation Turkish women).

## Additional file

Additional file 1:
**Index for assessment of the adequacy of prenatal care utilisation in the Dutch primary midwifery care context.**
