# Supplementary material for: Explanatory factors for first and second-generation non-western women’s inadequate prenatal care utilisation: a prospective cohort study
Source: BMC Pregnancy Childbirth. 2015 Apr 21;15:98. doi: 10.1186/s12884-015-0528-x (PMC4409999; doi:10.1186/s12884-015-0528-x)
Supplement: Additional file 1: — Index for assessment of the adequacy of prenatal care utilisation in the Dutch primary midwifery care context. [file 12884_2015_528_MOESM1_ESM.doc]

| **Additional file 1** Index for assessment of the adequacy of prenatal care utilisation in the Dutch primary midwifery care context (by A.W. Boerleider and E.I. Feijen - de Jong) | | | |
| --- | --- | --- | --- |
| Gestational age at birth | Gestational age at prenatal care entry | Number of prenatal visits | Categories |
| 0 weeks – < 12 weeks | < 12 weeks | ≥ 3 | 4 |
|  |  | 1-2 | 3 |
|  |  | 0 | 1 |
| 12 weeks – < 27 weeks | < 12 weeks | ≥6 | 4 |
|  |  | 3-5 | 3 |
|  |  | 2 | 2 |
|  |  | ≤1 | 1 |
|  | ≥ 12 weeks | * | 1 |
| 27 weeks – < 37 weeks | < 12 weeks | ≥10 | 4 |
|  |  | 6-9 | 3 |
|  |  | 4-5 | 2 |
|  |  | ≤ 3 | 1 |
|  | ≥ 12 weeks | * | 1 |
| 37 weeks – < 38 weeks | < 12 weeks | ≥ 13 | 4 |
|  |  | 10-12 | 3 |
|  |  | 6-9 | 2 |
|  |  | ≤5 | 1 |
|  | ≥ 12 weeks | * | 1 |
| 38 weeks – < 39 weeks | < 12 weeks | ≥ 14 | 4 |
|  |  | 10-13 | 3 |
|  |  | 6-9 | 2 |
|  |  | ≤5 | 1 |
|  | ≥ 12 weeks | * | 1 |
| 39 weeks – < 40 weeks | < 12 weeks | ≥ 15 | 4 |
|  |  | 11-14 | 3 |
|  |  | 7-10 | 2 |
|  |  | ≤6 | 1 |
|  | ≥ 12 weeks | * | 1 |
| 40 weeks – < 41 weeks | < 12 weeks | ≥ 16 | 4 |
|  |  | 12-15 | 3 |
|  |  | 7-11 | 2 |
|  |  | ≤6 | 1 |
|  | ≥ 12 weeks | * | 1 |
| 41 weeks – < 42 weeks | < 12 weeks | ≥ 17 | 4 |
|  |  | 12-16 | 3 |
|  |  | 8-11 | 2 |
|  |  | ≤7 | 1 |
|  | ≥ 12 weeks | * | 1 |
| *Ad* Irrespective of the number of visits*   1. Inadequate (gestational age at prenatal care entry ≥ 12 weeks *and/or* received < 50% of expected visits) 2. Intermediate (gestational age at prenatal care entry < 12 weeks *and received* 50%-79% of expected visits) 3. Adequate (gestational age at prenatal care entry < 12 weeks *and received* 80%-109% of expected visits) 4. Adequate Plus (gestational age at prenatal care entry < 12 weeks *and received ≥* 110% of expected visits) | | | |
